# Academic experiences, physical and mental health impact of COVID-19 pandemic on students and lecturers in health care education

**DOI:** 10.1186/s12909-021-02968-2

**Published:** 2021-10-27

**Authors:** Fazean Idris, Ihsan Nazurah Zulkipli, Khadizah Haji Abdul-Mumin, Siti Rohaiza Ahmad, Shahid Mitha, Hanif Abdul Rahman, Rajan Rajabalaya, Sheba Rani David, Lin Naing

**Affiliations:** PAPRSB Institute of Health Sciences, Universiti Brunei Darussalam, Jalan Tungku Link, Gadong, BE1410 Brunei Darussalam

**Keywords:** Online education, Experiences and impact, COVID-19, Pandemic, Students, Lecturers, University

## Abstract

**Background:**

In keeping with nation-wide efforts to contain the spread of COVID-19, Universiti Brunei Darussalam (UBD) transformed fully its pedagogical delivery to online mode, where we investigated teaching and learning experiences, physical and mental health of undergraduate students and lecturers during the COVID-19 pandemic.

**Methods:**

We conducted a cross-sectional study on undergraduate students and lecturers in a health science faculty using a self-developed pretested questionnaire through anonymous online data collection method.

**Results:**

Fifty-six lecturers (100% response rate) and 279 students (93.3% response rate) participated. The positive experiences reported by students include becoming independent (72.8%) and adapting to online learning (67.4%), while lecturers learned new teaching techniques (50.0%) and became more innovative (50.0%) by learning new tools (48.2%). However, studying at home caused students to feel more distracted (72.0%) with a feeling of uncertainty towards examinations (66.7%), while lecturers felt that students’ laboratory skills were compromised (44.6%). Even though online delivery of assessments enabled lecturers to explore all options (50.0%), they found it difficult to maintain appropriate questions (41.1%) and fair assessments (37.5%). Majority of students missed eating out (68.8%) and felt a lack of participation in extracurricular activities (64.9%), while lecturers reported more time for exercise (51.8%), despite having more screen time (50.0%) and computer-related physical stress (44.6%). In terms of mental health, increased stress in students was reported (64.9%), though they had more time for self-reflection (54.8%). Although lecturers reported a closer relationship with family (44.6%), they also felt more stressed due to deadlines, unexpected disruptions and higher workloads (44.6%) as well as concerns related to work, family and self (39.3%).

**Conclusion:**

In this abrupt shift to online teaching, students and lecturers in our study identified both positive and negative experiences including the impact on their physical and mental health. Our findings are important to provide the evidence for online pedagogical benefits and can serve to promote the enhancement and adaptation of digital technology in education. Our findings also aim to promote the importance of addressing physical and mental health issues of the university community’s well-being through provision of emotional and mental health support and appropriate programs.

## Introduction

Brunei Darussalam, a country with a population of 459,400, recorded its first imported case of COVID-19 on 9th March 2020 [1]. The government of Brunei Darussalam worked swiftly to prevent the spread of the virus, including closing of schools and allowing parts of the working population, including lecturers, to work from home. As the pandemic spread worldwide and the country saw a surge of positive cases, strict isolation measures were put in place to protect individuals from exposure to the virus while curbing the risk of community spread. In keeping with nation-wide efforts to contain the spread of the disease, Universiti Brunei Darussalam (UBD) moved its teaching and learning online for the duration of the ongoing semester to ensure that university students continued to receive their education [2].

With re-emerging surges in the pandemic, traditional on-site teaching and learning cannot be guaranteed on a sustainable basis. As social distancing is of vital importance at this stage to protect all communities, there has been a rapid pedagogical shift from traditional to online class sessions, face-to-face to virtual instruction, and seminars to webinars. The impact of the pandemic has brought on an era of radical technological transformation, with accelerated digitalization to the worldwide higher education system [3]. Thus, it is important for students and lecturers to utilize digital technology to their advantage as literature has suggested that online education is an effective and efficient learning environment, with benefits such as accessibility, opportunities for life-long-learning, improved quality and cost-effectiveness of educational resources [4]. Meanwhile lecturers can use this digital transformation period as a way to upgrade their skills while expanding capacity in new subject areas and providing a healthy balance between work and family [4]. Not only does online education offer a safe approach in avoiding the community spread of COVID-19 without the need of face-to-face interaction, it enhances student-centeredness, enabling lecturers to customize to the needs of students while offering flexibility in teaching delivery in terms of time and location for both parties [5]. Various online tools and digital technology are available for lecturers to adopt new technology and to equip them to be more innovative in their pedagogical delivery. Importantly, online education allows for a collaborative and interactive learning environment where educators can use a combination of audio, videos, and text to reach out to their students and to maintain their students’ engagement [5].

Research reporting the impact of COVID-19 on student education and welfare has observed that academic disruption can affect the university’s teaching delivery and result in psychosocial consequences for its university community. Students are often faced with increased anxiety during the pandemic, which may lead to decreased motivation towards studying [6] and this can be correlated with increased concerns on academic, social and economic well-being [7]. College students can struggle with loneliness and isolation not only because of disconnections from friends, but the abrupt disruption of the semester can cause cessation in their research projects and internships, leading to uncertainty in graduation and job market availability [6]. Students’ research output can also be markedly reduced due to the abandonment of hospital or clinical based research work [7].

COVID-19 while being a hazard to humanity has evolved institutions to invest in online learning, where Universiti Brunei Darussalam is no exception. Lecturers conducted teaching and assessments using multiple online learning platforms, such as Canvas, Microsoft Teams, Google Meet, Skype and Zoom while practical sessions were held with social and physical distancing, proper cleanliness, mask usage and through cohorting in a staggered approach following the Ministry of Health guidelines [8]. Soon, the advantages of online learning systems became apparent. Lectures and problem-based learning (PBL) could be continued using online learning management system, teleconferencing applications and devices. Teaching and learning activities in UBD continued undisrupted during the pandemic. With the implementation of online education during the pandemic, we wish to assess their impact by investigating the experiences of students’ learning as well as lecturers’ teaching and assessments, while exploring how this impacted their physical and mental health, since these two aspects will also be affected by the online mode of learning. Our study looks into both positive and negative experiences so to identify ways on developing the necessary procedures of online education implementation catered to the university community, while physical and mental health impact need to be explored to find ways of addressing any positive and negative effects that can be brought about from online education.

The implications of this study may guide the university to implement online learning as a necessary tool in their pedagogical delivery, regardless of the pandemic being over, to support the evidence that online education brings in bringing an effective and efficient learning environment. This is also seen as an innovative move and an investment towards digital technology, while highlighting the fundamentals of a pedagogical student-centered approach*.* In addition, this may benefit the local government authorities on how best to support online education in all schools and institutions by considering the positive and negative experiences of students and lecturers, while being prepared how this can affect their physical and mental health, as explored in this study.

## Materials and methods

### Research design

A cross-sectional study was conducted from 2nd April to 12th April 2020 amongst undergraduate students and lecturers involved in undergraduate teaching in the Bachelor of Health Science programmes (majoring in Medicine, Dentistry, Pharmacy, Biomedical Sciences, Nursing & Midwifery) from the Pengiran Anak Puteri Rashidah Sa’adatul Bolkiah Institute of Health Sciences (PAPRSB-IHS), Universiti Brunei Darussalam.

### Sampling

The undergraduate students were those who had registered for at least one module during the January to May 2020 semester. Students in community outreach, internships or study-abroad programmes were excluded from the study. All 299 undergraduate students and 56 lecturers of the eligible population were invited to participate in the study by the Assistant Executive Officer of the Institute, who acted as gatekeeper of the study. The invitation was sent out via email to the students’ mass email at student.ihs@ubd.edu.bn and lecturers’ email at staff.ihs@ubd.edu.bn. Those who agreed to participate were free to click on the link given in the email invitation, which brought them to the participant information sheet and consent form.

### Instrument

The questionnaire was designed after three mini-workshop sessions, two of which were held with students and one with area experts, i.e., lecturers from each health science major (namely Medicine, Dentistry, Pharmacy, Biomedical Sciences, Nursing & Midwifery). Questionnaire was designed based on the discussions generated from the workshop participants as well as input from the research team to ensure content validity. The questionnaire was pretested among a small group of lecturers and students to ensure comprehensibility before it was finalized. These questions were relating to their teaching and learning experiences as well as their perceived physical and mental health during the COVID-19 pandemic. For lecturers, an additional section on their opinions of positive and negative effects of assessments was included in the questionnaire. Minor corrections to wordings and format were made to the final questionnaire following pretesting. Students and lecturers invited to participate in the main study were informed in the invitation email that they did not have do so had they been a part of the pre-test.

### Data collection

The questionnaire was set up on the *Qualtrics* online platform subscribed by the University to collect the data. A uniform link was created and sent to the participants at their respective group emails to ensure anonymous data collection, which led to the participant information sheet and consent form. Participants were reminded about the study twice via mass email after 3 and 7 days. Participants’ individual responses from the questionnaires were non-identifiable, as these had been received anonymously through the Qualtrics online platform.

### Ethical considerations

The ethical approval for the project was granted by PAPRSB-IHS Research Ethics Committee and University Research Ethics Committee of Universiti Brunei Darussalam (UBD/OAVCR/UREC/Apr2020–01).

### Data analysis

Data was downloaded into Microsoft Excel from the *Qualtrics* platform and was processed for labeling categories and missing data. Descriptive statistics using count and percentage were used to describe demographic characteristics and study factors including online learning and teaching experiences, designing and providing assessment, and impact on mental health for students and lecturers. Bar graphs were also used to illustrate relevant study factors. Chi-square test was used to investigate association between demographic characteristics and study factors, ensuring assumptions such as minimum expected count was met prior to applying the analysis*. P*-value of less than 0.05 was considered as statistically significant. R (version 4.0.2) and RStudio (version 1.3.1056) for Windows were used for all analyses.

## Results

### Demographic characteristics of participants

A total of 279 health science undergraduate students (93.3% response rate) and 56 lecturers (100% response rate) participated in this study. Amongst the students, 103 (36.9%) were from programmes of nursing, 60 (21.5%) from medicine, 50 (17.9%) from biomedical sciences, 36 (12.9%) from pharmacy and 30 (10.8%) from dentistry. 72.6% of students were female with majority of them in Year 1 (38.9%), followed by Year 2 (31.3%), Year 3 (18.4%) and Year 4 (11.5%). Over 95% of students reported having internet access*.* 16 (28.6%) of lecturers were from programmes of nursing, 12 (21.4%) from biomedical sciences, 8 (14.3%) from medicine, 4 (7.1%) from pharmacy and 2 (3.6%) from dentistry. Majority (66.7%) of lecturers were female, local Bruneians (78.6%), aged between 41 to 50 years old (43.9%) and had more than 10 years of academic experience (51.2%).

### Positive and negative online learning experiences of students

A majority of students reported positively that online learning made them more independent and that they could adapt to online learning and its sudden changes. However, studying at home also made a majority of students more distracted and the online mode made them feel uncertain about assessment and examination while having a lack of spontaneous interaction with their lecturers. Details of the students’ positive and negative experiences of learning during COVID-19 pandemic are illustrated in Table 1.

**Table 1:** Positive and negative online learning experiences of students (*n* = 279)

Learning experience	Total (***n =*** 279)	Biomed. (***n*** = 50)	Dentistry (***n =*** 30)	Medicine (***n*** = 60)	N&MW (***n*** = 103)	Pharmacy (***n*** = 36)	***p***- value^**a**^
	***n*** (%)	***n*** (%)	***n*** (%)	***n*** (%)	***n*** (%)	***n*** (%)	
**Positive**							
Be independent	203 (72.8)	39 (78.0)	25 (83.3)	40 (66.7)	77 (74.8)	22 (61.1)	0.087
Adapt online learning	188 (67.4)	35 (70.0)	25 (83.3)	40 (66.7)	70 (68.0)	18 (50.0)	0.057
Adapt to sudden change	173 (62.0)	29 (58.0)	23 (76.7)	40 (66.7)	60 (58.3)	20 (55.6)	0.374
Set and keep schedule	164 (58.8)	27 (54.0)	18 (60.0)	32 (53.3)	69 (67.0)	18 (50.0)	0.366
Be self-motivated	161 (57.7)	30 (60.0)	20 (66.7)	32 (53.3)	59 (57.3)	20 (55.6)	0.658
More attentive online	138 (49.5)	22 (44.0)	16 (53.3)	22 (36.7)	53 (51.5)	17 (47.2)	0.420
Environmentally friendly	131 (47.0)	28 (56.0)	13 (43.3)	17 (28.3)	59 (57.3)	14 (38.9)	0.002
Replay recorded lectures	128 (45.9)	29 (58.0)	9 (30.0)	23 (38.3)	55 (53.4)	12 (33.3)	0.007
Lecturers more interaction	79 (28.3)	15 (30.0)	7 (23.3)	8 (13.3)	39 (37.9)	10 (27.8)	0.018
**Negative**							
Distracted when working from home	201 (72.0)	34 (68.0)	28 (93.3)	40 (66.7)	74 (71.8)	25 (69.4)	0.103
Less certain about assessment and exam	186 (66.7)	34 (68.0)	27 (90.0)	41 (68.3)	62 (60.2)	22 (61.1)	0.030
Live lectures distracted or noisy background	155 (55.6)	28 (56.0)	22 (73.3)	26 (43.3)	63 (61.2)	16 (44.4)	0.031
No spontaneous interaction	155 (55.6)	27 (54.0)	23 (76.7)	28 (46.7)	61 (59.2)	16 (44.4)	0.052
Group work become challenging	143 (51.3)	23 (46.0)	18 (60.0)	22 (36.7)	61 (59.2)	19 (52.8)	0.068
Missed live lectures frequently	89 (31.9)	13 (26.0)	12 (40.0)	9 (15.0)	46 (44.7)	9 (25.0)	0.002
Lecturers less prompt to answer online questions	80 (28.7)	11 (22.0)	9 (30.0)	14 (23.3)	41 (39.8)	15 (41.7)	0.027

^a^Chi-square test for independence; Biomed. = Biomedical Sciences; N&MW=Nursing and Midwifery

### Positive and negative teaching experiences of lecturers

**Fig. 1:**
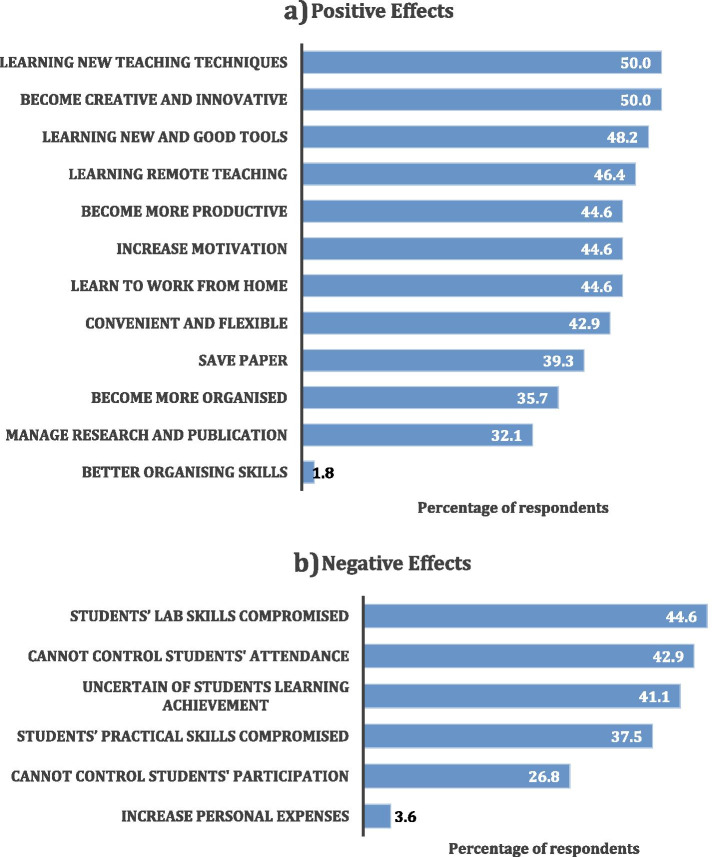
**a** Positive and **b** Negative teaching experiences of lecturers (*n* = 56)

A majority of lecturers identified their experience of online teaching as a way of learning new teaching techniques and becoming more creative by learning new and good tools. However, on the downside, lecturers felt that students’ laboratory skills may have been compromised and could not control students’ attendances. They were also uncertain about students’ learning achievements. Full responses of the lecturers’ positive and negative experiences of teaching including other academic activities during the study are illustrated in Fig. 1.

### Positive and negative experiences of lecturers on designing and providing assessments

**Fig. 2:**
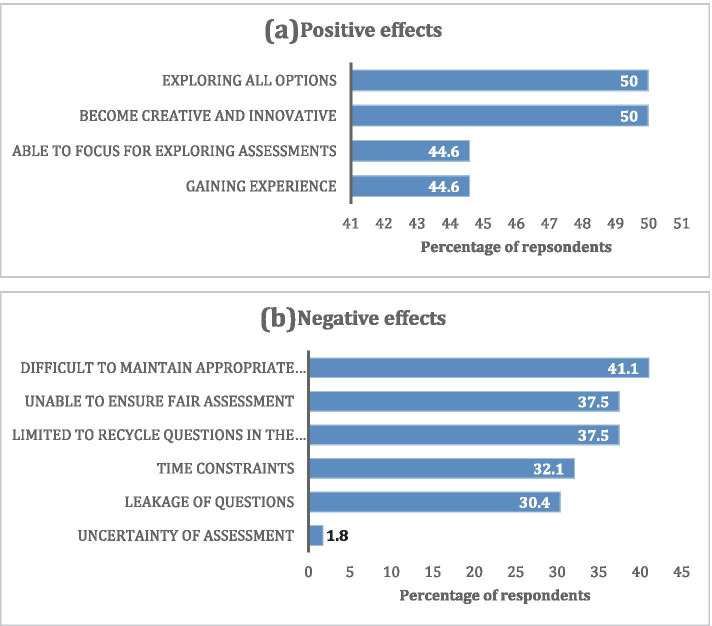
**a** Positive and **b** Negative experiences on lecturers’ designing and providing assessments (*n* = 56)

While online design and delivery of assessments made half of lecturers able to explore all options while becoming creative and innovative, they found it difficult to maintain appropriate questions and fair assessments for the students. Figure 2 further illustrates lecturers’ positive and negative experiences of designing and providing assessments in this study,

### Physical and mental health impact on students

Majority of students reported that they missed eating outside and experienced a lack of extracurricular activities, with only 35.8% of them performing exercise at home. They also reported more back problems and eye strain or dry eyes. In terms of their mental health, they reported more stress, followed by anxiety, loneliness and depression, but that this time away from university enabled more time for self-reflection. Table 2 shows the physical and mental health experiences of students in this study.

**Table 2:** Physical and mental health experiences of students (*n* = 279)

Reported Health Experiences	Total (***n =*** 279)	Biomed. (***n*** = 50)	Dentistry (***n =*** 30)	Medicine (***n*** = 60)	N&MW (***n*** = 103)	Pharmacy (***n*** = 36)	***p***-value^**a**^
	***n*** (%)	***n*** (%)	***n*** (%)	***n*** (%)	***n*** (%)	***n*** (%)	
**Physical Health**							
Miss eating outside	192 (68.8)	32 (64.0)	25 (83.3)	41 (68.3)	70 (68.0)	24 (66.7)	0.580
No more ECAs	181 (64.9)	34 (68.0)	24 (80.0)	36 (60.0)	64 (62.1)	23 (63.9)	0.234
Home cooked meals	169 (60.6)	35 (70.0)	23 (76.7)	29 (48.3)	63 (61.2)	19 (52.8)	**0.016**
Back problems	163 (58.4)	30 (60.0)	22 (73.3)	29 (48.3)	61 (59.2)	21 (58.3)	0.190
Eye strain/dry eyes	143 (51.3)	24 (48.0)	22 (73.3)	25 (41.7)	56 (54.4)	16 (44.4)	0.066
Less time for exercise	125 (44.8)	17 (34.0)	15 (50.0)	22 (36.7)	56 (54.4)	15 (41.7)	0.144
Gained weight	116 (41.6)	18 (36.0)	13 (43.3)	18 (30.0)	52 (50.5)	15 (41.7)	0.150
More exercise at home	100 (35.8)	23 (46.0)	15 (50.0)	19 (31.7)	29 (28.2)	14 (38.9)	**0.043**
**Mental health**							
More stressed	181 (64.9)	37 (74.0)	19 (63.3)	30 (50.0)	74 (71.8)	22 (61.1)	**0.010**
Self-reflection	153 (54.8)	28 (56.0)	22 (73.3)	34 (56.7)	51 (49.5)	18 (50.0)	0.187
Anxiety problem	138 (49.5)	25 (50.0)	12 (40.0)	25 (41.7)	61 (59.2)	15 (41.7)	0.115
Felt lonely	131 (47.0)	25 (50.0)	18 (60.0)	35 (58.3)	41 (39.8)	12 (33.3)	**0.033**
More relaxed	127 (45.5)	21 (42.0)	19 (63.3)	28 (46.7)	43 (41.7)	16 (44.4)	0.357
More depressed	95 (34.1)	13 (26.0)	11 (36.7)	18 (30.0)	38 (36.9)	15 (41.7)	0.604

^a^ Chi-square test for independence; Biomed. = Biomedical Sciences; N&MW=Nursing and Midwifery

### Physical and mental health impact on lecturers

**Fig. 3:**
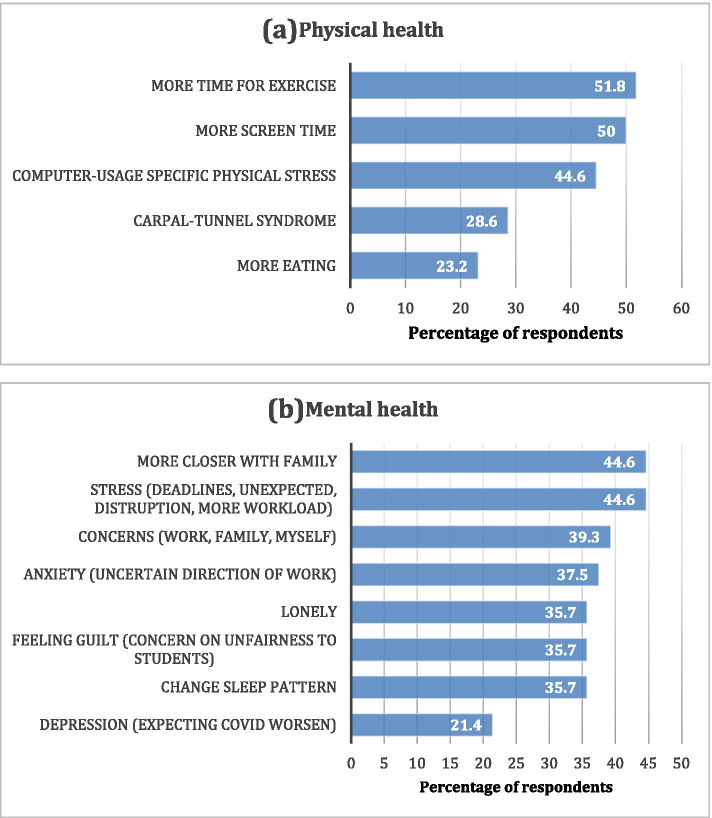
**a** Physical and **b** Mental health experiences of lecturers (*n* = 56)

Meanwhile, the lecturers had more time for exercise but yet the increased screen time led to computer-related physical stress (Fig. 3a). Although the lecturers reported a closer relationship with family, they admitted to being stressed due to deadlines, unexpected disruptions and higher workload, with concerns related to work, family and self (Fig. 3b). Fortunately, there was a low percentage of reported depression. Figure 3 shows the reported responses of lecturers from the impact of the pandemic on their physical and mental health.

## Discussion

### Students’ experiences from online education

Our study provides favorable evidence of online learning among students during the pandemic with a majority reporting that they became independent, adapted to online learning and became more self-motivated, reinforcing that online pedagogy certainly has its benefits in encouraging students to work independently and enhance their self-efficacy. Evidence has suggested that students who are independent learners, work to higher standards, use a range of strategies in their learning, are competent in their problem-solving skills, more motivated and have higher self-esteem [9]. In addition, such pedagogical method can enhance students to become self-directed learners, which is an important competency required to be a life-long learner, particularly in the field of health care [10].

On the contrary, the downside reported by the students in our study was distractions when studying from home, such as a noisy background during live video lectures. This adds to the current knowledge on the dilemma of home learning. Environmental factors at home may be inevitable due to conflict in tasks with either daily household chores and family or personal commitments at home. In addition, students’ family or household members may not have appreciated the privacy and quietness that students need to focus when receiving online education. Students may also be challenged by their academic roles as students, which are in conflict with their roles as family members to perform assigned family tasks and duties during the pandemic.

Another negative experience identified by students was the lack of spontaneous interaction with lecturers. Although online learning signified promising advances in technology, such platforms may limit engagement between them and efforts should be made to humanize the learning process as much as possible. Thus, lecturers should plan effective strategies for giving online instructions to facilitate feedback and encourage students to ask questions, such as to constantly remind and prompt their students to participate in online discussions and quizzes and to make the online sessions dynamic, interesting, and interactive [5]. Other suggestions include regular reminders for students to make them alert and attentive, through use of social media, text messages and various messaging applications to personally communicate with students. It is important to engage in interactions with students to plan their learning activities, assist in carrying out self-assessments and increase their confidence in solving problems [11] as research has documented that interaction between students and lecturers as well as university’s provision of emotional and social support are essential components for effective learning [12]. Certainly, lecturers who demonstrate a genuine personal and academic interest in students report stronger student outcomes [13], while regular two-way feedback can enhance self-efficacy and motivation [14].

### Lecturers’ experiences from online education and assessments

Findings from the lecturers highlighted similar sentiments to the students that online learning modalities were an effective source of teaching and learning, with lecturers reporting that they were learning new teaching techniques while becoming creative and innovative. With the university closure, modifications to clinical skills teaching and placements had to be made, where student placements in clinical settings was forced to cease as a crucial move to allow clinicians to focus on managing patients affected by the pandemic and limit exposure of students to health risks in hospitals. This was a disadvantage for students as patient contact is necessary for their learning and assessment. In a London-based medical school, face-to-face lectures and clinical-based teaching during the pandemic were substituted by giving medical students access to an online repository of patient interview recordings and cases with tele-teaching technologies [15]. Such a modality would demand great efforts in interactions from both students and lecturers and if compromised, this may result in a decrease in quantity and quality of the discussions [16]. As our study had identified that hands-on session such as laboratory and clinical skills teaching were limitations of online learning, lecturers utilized online simulated patients, role-plays, sharing recorded videos of laboratory and clinical skills demonstration to teach history taking, clinical reasoning and communication skills. Despite such efforts, our findings demonstrated lecturers’ concerns that students’ practical skills may have been compromised. Lecturers may also have limited confidence in transitioning to online clinical teaching as well as a limited experience in interactive methods using digital technologies; hence they feel uncertain about students’ achievement of learning outcomes.

Like other universities worldwide, abrupt transitions to the assessments of students had to be made in midst of the pandemic. Assessments were also converted to online methods, making use of online learning management systems for written assignments, oral presentations and vivas. Half of the lecturers from our study reported positively that this transition allowed them to explore options of online examinations, including the opportunity to become more innovative in their approaches, was viewed as a positive but steep learning curve for them. Lecturers were also prepared for the online mode of examinations through a series of workshops to familiarize themselves with the learning management system and to learn various modalities offered for question setting, and marking or grading strategies. However, lecturers also reported the difficulty to maintain appropriate questions and ensure fair assessments while a majority of students felt uncertain about their assessments. During this transition, there was a need to reduce the width and depth of assessments in response to the premature exclusion of some learning objectives due to the pandemic. Clinical assessments had to be substituted with online viva describing the skills in absence of models, patients, instruments or simulation. In addition, lecturers were concerned about achieving fair assessments where exams were proctored online, giving rise to a potential compromise in academic integrity. Another concern raised was loss of internet connectivity during the online examinations leading to a period of non-invigilation of the student as well as whether duration was appropriately allocated to attempt the examinations through this online proctoring. Despite mock tests and dry runs, lecturers had to prepare to allow more time to compensate for such potential issues.

The British Medical Association [17] published a document outlining a set of principles in education of medical students during COVID-19, which was set to ensure their fair assessment and progression, and to consider reasonable adjustments for students with poor access to internet or computer and at disadvantaged locations (i.e. in rural areas) as well as test securities. In the UK, medical schools have been advised to make alternative arrangements to reschedule their exams in a timely manner and to utilize a variety of resources for teaching and assessments. Provision of facilities and computers as well as a clear policy on appeals such as when assessment had to be terminated due to a technical failure, were drawn up. Other universities may employ different approaches such as canceling or rescheduling exams, using modified format exams (online or onsite segregated groups) and using “expert” provided grades. Fortunately, the Teaching and Learning Centre in UBD, continued to provide online training workshops for lecturers to increase their confidence in utilization and delivery of various online technologies in teaching.

### Physical health impact of online education on students and lecturers

The global outbreak of COVID-19 resulted in closures of sports complexes and venues, limiting active participation in sports activities subjecting individuals to be less physically active with more screen time, and consuming unhealthy diets, which may lead to a health issue. Student respondents in our study reported ‘missing eating outside’ and ‘a lack of participation in extracurricular activities’, with ‘more back problems’ and ‘eye strain/dry eyes’. Meanwhile, more than half of the lecturers reported having ‘more time for exercise’ although they had ‘more screen time’ leading to ‘computer related physical stress’ such as back problems, dry eyes or strains and carpal tunnel syndrome. These findings concur with the social distancing measures implemented in Brunei Darussalam during the pandemic, where food premises were not allowed to provide dine-in services. University events and extracurricular activities were also cancelled during the study period. The increased need of computers or devices for online learning led to physical effects as noted in our study, such as back problems, eye strain, dry eyes, computer-related physical stress, most likely to be exacerbated with prolonged screen time.

In an online survey looking into exercise and social behaviors conducted by University College London in 2020, 85% of survey respondents did not engage in any moderate or strenuous exercise while 40% did not do any gentle exercise such as going for a walk [18]. This was especially seen in the respondents in that survey of ages 18–30 years, where 4 out of 5 did not engage in any moderate or strenuous exercise. The same survey reported that respondents aged above 30 years old were more engaged in gentle activity and concerned about their health in view of their higher risk of lifestyle diseases. In Brunei Darussalam, the government imposed a semi-lockdown control during the pandemic which still allowed individuals to go out as long as they followed protocols of limited numbers and only to do so for essential needs. This may be the likely explanation concerning the lecturers in our study who may have greater self-awareness and responsibility of health issues and hence the need to be physically active at home through exercises or housework. While online exercise resources such as physical strength training may be more feasible to older participants, younger people may prefer participation in social or group sports, which at that time, had been restricted. Such findings are consistent with reports that a reduction in physical activity and increase in screen time during the pandemic negatively impacted individual’s physical and mental health [19].

### Mental health impact of online education on students and lecturers

Social distancing following school closures may increase mental health problems in adolescents at a time when they are experiencing anxiety over the pandemic, which may be worsened by concerns on future employment [20], the absence of interpersonal communication [21] and lack of understanding of the virus transmission in addition to fearing the unknown. In our study, students identified the feelings of stress, anxiety, loneliness and depression as the mental health effects of the pandemic. Students may experience stress due to increased pressure to perform independent learning and abandoning their usual routines, which can lead to psychological consequences such as anxiety, depression, difficulty sleeping and stress eating [22]. As clinical placements had to be cancelled, this could exacerbate students’ loneliness, in addition to setting apart from their usual social contacts in schools or colleges [23]. Such findings seen in our study are not dissimilar to other studies. A study in China reported that university students showed higher anxiety levels as students faced the new term with fully online learning [20] while French university students experienced anxiety and stress during social isolation [24]. Anxiety and stress have been observed to affect learning skills such as time management, concentration, study motivation and learning methods and which can affect students’ performance [25] and potentially poor academic progression [7].

Similarly, mental health impacts were also observed among lecturers where almost half of them in our study felt more stressed due to meeting deadlines and unexpected distractions. Concerns related to work, family and self may have also compromised mental health. On the other hand, our findings reported that lecturers felt a closer relationship with their families. Interestingly, when compared to students, lecturers experienced less stress, anxiety and loneliness. Our findings can be compared to a study in Spain where university staff reported lower scores of depression, anxiety and stress using the DASS-21 tool compared with students [26]. As the older working community has been working professionally under stressful conditions and able to adjust with work protocols and changes, they may be better tolerant in handling stress more than students, who make up a vulnerable group of adolescents. Being closer to family and gaining their support at home also plays an important role to alleviate stressors amongst the lecturers.

PAPRSB-IHS in UBD is committed to developing the role of the university as an advocate for health. This includes increasing the health promotion and education content in their teaching and research curriculum by offering a number of health education modules for students from non-health science faculties, while students in the health science programmes of Medicine, Dentistry, Pharmacy, Nursing and Midwifery conduct community outreach projects related to health by establishing alliances with multiple health agencies. The Institute is also in the process of establishing the university clinic, which aims to promote primary health care and manage specific health problems of both university and public communities, as well as being an advocate for health in the community by creating partnerships with primary health care and welfare agencies within and outside the university. This can also be achieved by developing health promotion links to support health development in the community and increasing the profile of health, health promotion and public health issues in teaching and research [27].

It is important to promote the well-being of the university community by integrating health into the culture, structures and processes of the university through formation of healthy working, learning and living environments for students and staff, particularly during the pandemic where social distancing should not mean social isolation. To be a health-promoting university, promotion of sustainable health policies and planning throughout the university are required to provide healthy working and supportive social environments [27]. This includes providing welfare, medical and health-related support services sensitive to the needs of students and staff during times of pandemic when physical use of social, leisure, sports and cultural facilities are affected. The university, through the Safety, Health and Environment office, has ensured steps to promote physical and mental health awareness during this time through sharing of resources and constant communication regarding work scheduling, reminding all to be connected and well-informed, staying active through app-based workouts or online exercises, and engaging in activities that benefit one’s well-being.

### Limitations

A few limitations were identified with this study. Although we received a 100% response rate from lecturers, only 93.3% of students participated in the study. Some shortcomings with the questionnaire are also expected from our study as the design and pretesting of questionnaire was done in a relatively short time when ideally it would be better to run the pretest a few more times. As this is a perception-based questionnaire, responses were based on the answers suggested in the questionnaire and may introduce some degree of subjectivity. To overcome the limitations, we have also conducted a qualitative study in the form of focus group discussions to identify in depth the experiences and challenges of online education in terms of teaching, learning, assessments as well as physical and mental health, amongst various groups from each programme within the faculty, for both students and lecturers. Results of the study will be published in due course.

## Conclusion

A majority of students from our study reported positively that online learning made them more independent and that they could adapt to online learning with its sudden changes, while lecturers identified the experience of online teaching as a way of learning new teaching techniques and becoming more creative by learning new and good tools. However, studying at home caused students to feel more distracted and they identified a lack of spontaneous interaction with their lecturers. Lecturers felt that students’ laboratory skills may have been compromised and were uncertain about students’ learning achievements. While online delivery of assessments enabled lecturers to explore options while becoming creative and innovative, they found it difficult to maintain appropriate and fair assessments, for which the students were also uncertain about. Majority of students missed ‘eating out’ and felt a lack of participation in extracurricular activities, with more back and eye problems. Although lecturers reported they had more time for exercise, the increased screen time led to computer related physical stress, such as back problems, dry eyes or eye strains and carpal tunnel syndrome.

Thus, we can identify that COVID-19 pandemic has brought many changes to academia, many of them perceived as being positive with few challenges, which can be overcome by promoting effective digital transformation through greater quality of innovative pedagogical methods, perhaps using a blended pedagogy approach of physical and online classrooms that may be preferred by both students and lecturers to address some of the challenges experienced with online education. This can be achieved by upgrading technological infrastructure through provision of various technological resources suitable for the students and lecturers’ needs allowing for more online student-lecturer interaction. To address the issue of assessment challenges, the university has converted most of their examinations into coursework, making them more appropriate and fairer to assess. At the same time, to ensure a sustainable and continued familiarity of online tools, it is important to cultivate a digital learning culture amongst the university community, including the administration and support staff while ensuring all have equal access to the technological resources needed [28]. Such approaches will require faculty development to improve the university community’s readiness and confidence in utilizing digital technology responding to tools allowing for more interaction and better assessments, as well as policies highly encouraging the use of online or blended technological approaches in delivering teaching and assessments. In addition, it is important to promote the well-being of the university community by implementing healthy policy and health promotion interventions within the university that emphasize on creating healthy working, learning and living environments for students and staff, particularly tailoring to the specific physical and mental health issues as observed in this study. Further studies on the quantitative evaluation of the implementation of the online studies on impact on physical and mental health through appropriate measurements would be beneficial to explore the relevant benefits.

## Data Availability

The datasets used and/or analysed during this study is available from the corresponding author on request.

## Abbreviations

UBD: Universiti Brunei Darussalam
PAPRSB-IHS: Pengiran Anak Puteri Rashidah Sa’adatul Bolkiah Institute of Health Sciences
PBL: Problem-based learning
